# Apoptotic Cells Induce NF-κB and Inflammasome Negative Signaling

**DOI:** 10.1371/journal.pone.0122440

**Published:** 2015-03-30

**Authors:** Amir Grau, Adi Tabib, Inna Grau, Inna Reiner, Dror Mevorach

**Affiliations:** The Laboratory for Cellular and Molecular Immunology, Rheumatology Research Center, Department of Medicine, Hadassah-Hebrew University Medical Center, Jerusalem, Israel; Virginia Tech, UNITED STATES

## Abstract

As they undergo phagocytosis, most early apoptotic cells negatively regulate proinflammatory signaling and were suggested as a major mechanism in the resolution of inflammation. The dextran sulfate sodium model is generally viewed as an epithelial damage model suited to investigate innate immune responses. Macrophages primed with LPS and subsequently exposed to DSS secrete high levels of IL-1β in an NLRP3-, ASC-, and caspase-1-dependent manner. The aim of this research was to test the therapeutic effect of a single dose of apoptotic cells in a DSS-colitis model and to explore possible mechanisms. Primary peritoneal macrophages, the DSS mice model, and *Nlrp3*-deficient mice, were used to assess the effect apoptotic cells on colitis. Immunohistochemistry, flow-cytometer, and western blots helped to explore the effect and mechanisms. Using a variety of NLRP3 triggering mechanisms, we show that apoptotic cells negatively regulate NF-κB and NLRP3 activation in primary peritoneal macrophages, at pre- and post-transcription levels, via inhibition of reactive oxygen species, lysosomal stabilization, and blocking K+ efflux. This property of apoptotic cells is demonstrated in a dramatic clinical, histological, and immunological amelioration of DSS colitis in Balb/c and B6 mice following a single administration of apoptotic cells.

## Introduction

Even as they recruit phagocytes and undergo phagocytosis, most apoptotic cells negatively regulate proinflammatory signaling and were suggested as a major mechanism in the resolution of inflammation [1–3]. Several anti-inflammatory patterns have been identified, and apparently multiple mechanisms of immune suppression could be used during apoptotic cell death and the clearance of apoptotic cells [4–6]. Inhibition of NF-κB by apoptotic cells has been shown by others [7,8] and by our team [9], and there is evidence that nuclear migration of p65 is inhibited at the transcriptional or post-transcriptional level. Mer receptor tyrosine kinase (MerTK) was also found to activate the phosphatidylinositol 3-kinase (PI3K)/AKT pathway, which negatively regulates NF-κB [10]. NF-κB is a major transcription factor that has been implicated as a critical regulator of gene expression in the setting of inflammation in general, particularly in IL-1β production and secretion [11]. However, more specific mechanisms of inhibition that could explain resolution of inflammation are still lacking. Inhibition of transcriptional factors could explain inhibition of de novo pro-IL-1β production, but does not clearly explain post-transcriptional IL-1β production and secretion.

Inflammatory bowel diseases (IBD) are characterized by chronic intestinal inflammation with dysregulation of the mucosal immune system manifested as Crohn’s disease and ulcerative colitis [12]. The etiology of IBD is incompletely understood with both genetic and environmental factors contribute to the dysregulation of the mucosal immune system in the gastrointestinal tract. Genetic factors [13], and environmental factors that include both intestinal microflora [14], and danger signals such as dextran sodium sulfate (DSS) were all shown to induce intestinal inflammation. TNFα and IFNγ blockade [13] and anti-IL-1β strategies, as well as antibiotic treatment [15] were able to ameliorate colitis induction, suggesting a role for nuclear factor-kappa B (NF-κB) and inflammasome inhibition of macrophages and dendritic cells in the lamina propria.

In the present study we were interested to examine whether apoptotic cells negatively signal the innate immune system and ameliorate colitis in an IBD model; DSS-induced colitis, and to further explore a possible mechanism. Here we show that single infusion of apoptotic cells clearly ameliorate clinical and histological colitis via inhibition of NF-κB and NLRP3 inflammasome and thus, represent a novel therapeutic approach.

## Materials and Methods

### Animals

BALB/c or C57BL/6 mice were obtained from Harlan Inc. (Jerusalem, Israel). *Nlrp3*-deficient mice were described elsewhere [16] and were a generous gift of Jürg Tschopp via Luke O'Neill (Trinity College, Dublin, Ireland). All experiments were performed in the specific pathogen-free unit of the Hebrew University—Hadassah School of Medicine (Jerusalem, Israel). The study protocol was approved by the Hebrew University Ethics Committee for Animal Experiments and all efforts were made to minimize suffering.

### Cell cultures and reagents

Cells were cultured in Dulbecco’s modified Eagle’s medium (DMEM), with high glucose supplementation (Invitrogen-Gibco, Carlsbad, CA), and with 1% L-glutamine (Biological Industries, Israel), 10% fetal bovine serum (Biological Industries), and 10 μg/mL ciprofloxacin (Sigma Aldrich, Israel). The caspase-1 inhibitor z-YVAD-fmk, nigericin, and bafilomycin A1 were purchased from Calbiochem (Darmstadt, Germany). N-acetyl-L-cysteine (NAC) and lipopolysaccharide (LPS) were from Sigma Aldrich. DSS reagent was from MP Biomedicals (Illkirch, France). For immunostaining, the following antibodies were used: anti-COX2 (Cayman Chemicals, Ann Arbor MI, USA), anti-myeloperoxidase (Thermo Scientific, Waltham MA, USA), anti-phospho-IκBα and anti-phospho-NF-κB p65 (Cell Signaling, Danvers MA, USA).

### Generation of apoptotic cells

Irradiated (4000 rad) apoptotic splenocytes syngeneic cells or human apoptotic mononuclear cells isolated by leukopheresis and induced using methylprednisolone (ApoCell), were used. Apoptosis was verified using Annexin V and propidium iodide (PI) (MBL International, Woburn MA, USA). ApoCell contained >60% Annexin- and <5% PI-positive cells.

### Isolation of peritoneal macrophages and co-housing

Primary resident peritoneal macrophages (pMΦ) of WT or *Nlrp3* ^*-/-*^ mice were generated as described elsewhere [17]. Mice were sacrificed under isoflurane anesthesia by cervical dislocation, injected intraperitoneally with 10 mL of PBS, and peritoneal lavage was then performed. Peritoneal lavage fluid was centrifuged and plated into culture dishes for 2h. Cells were washed, and adherent cells were used for cytokine assays. Where indicated, experiments were preformed after 4 weeks of co-housing WT and *Nlrp3*-deficient mice to neutralize the microbiota effect [14].

### IL-1β ELISA

pMΦ were seeded into 96-well plates at a density of 2x10^5^ cells per well. After LPS priming for 1 h, cells were stimulated with different activators for 24 h. Cell culture supernatant was used for ELISA (Biolegend, San Diego CA, USA), which was performed according to the manufacturer’s protocol.

### Western blotting

The processed IL-1β p17 subunit and activated caspase-1 p10 subunit and their releases into the culture supernatant were determined by Western blotting. In brief, 12 hours after indicated activators addition, macrophages were lysed in lysis buffer (50mM Tris-HCl pH8.0, 5mM EDTA, 150mM NaCl, 1% Triton-X 100 and a protease inhibitor cocktail (Roche)) and stored at -80°C until analyzed. The supernatant was collected and suspended in SDS-PAGE sample buffer, and heated to 85°C for 10 min. Protein from 1x10^6^ macrophages was loaded per well of a 15% acrylamide gel and transferred to a PVDF (poly(vinylidene difluoride)) membrane by electroblotting. Western blots were performed with anti–mouse IL-1β antibody (clone B122; Biolegend) diluted 1:500 and anti-mouse caspase-1 p10 antibody (Santa Cruz) diluted 1:1000. Appropriate HRP-conjugated secondary antibodies (Jackson ImmunoResearch Laboratories, West Grove, PA, USA) were used and proteins detected using ECL reagent (Biological Industries). An anti-mouse actin served as a loading control.

### Real-time polymerase chain reaction (RT-PCR)

After animal scarification, a 0.5 cm of distal colon was snap-frozen in liquid nitrogen and total RNA was isolated by TRI-reagent (sigma). Two micrograms of total RNA was transcribed using the high-capacity cDNA synthesis kit (Applied Biosystems). cDNA corresponding to 20 ng of total RNA was used as a template in the PCR consisting of ABI MasterMix (Applied Biosystems, Darmstadt, Germany) and predesigned TaqMan gene expression systems (Applied Biosystems) according to the manufacturer’s instructions. PCR was performed using 7900HT Fast Real-Time PCR System (Applied Biosystems). For detection of IL-1β mRNA, primers Mm01336189_m1 (IL-1β) were used. Samples were assayed in triplicate and the mRNA expression was displayed as a normalized ratio of the target mRNA to hypoxanthine guanine phosphoribosyltransferase (HPRT Mm01545399_m1) and glucuronidase-beta (GusB Mm00446956_m1), all primers from Applied Biosystems.

### Measurement of reactive oxygen species (ROS)

Production of ROS by DSS was measured with the ROS detection kit (Enzo Life Sciences, Farmingdale NY, USA) according to the manufacturer’s protocol. pMΦ from female B6 mice were seeded onto eight-chamber slides at density 0.1x10^6^ cells/chamber, and cultured at 37°C for 24 h. Thereafter, pMΦ were washed twice with PBS, treated for 2 h with apoptotic cells, washed, and treated with 3% DSS for an additional 30 min. Negative control cells were treated with media only. After washing, the cells were suspended in 200 μl of DMEM and stained with the ROS detection reagent (1μM) for 30 min. DSS-induced intracellular ROS was detected by fluorescence microscopic (Eclipse E400, Nikon, Tokyo, Japan) examination at 488 nm excitation wavelength with a 525-nm emission filter. (Original magnification x100). Where flow cytometry detection was applied, MΦ were detached by trypsin-EDTA after treatment, washed, and analyzed using an LSRII instrument (BD Biosciences).

### Lysosomal stability evaluation

Lysosomal damage by DSS challenge was evaluated by acridine orange stain as described elsewhere [17]. Briefly, macrophages were plated into 24-well culture dishes overnight and introduced to apoptotic cells (1:8) for 2 h. Macrophages were then washed, primed with LPS, and stimulated for 24 h with DSS. Cells were then washed and incubated with 0.25μg/mL acridine orange for 15 min for lysosome stain. Lysosomal damage was determined as loss of fluorescence intensity emission at 600–650 nm with an LSRII (BD Biosciences). Data analysis was performed using FCS express v3 software (De Novo Software).

### Confocal microscopy

Evaluation of lysosomal rupture was visualized by confocal microscopy. pMΦ were plated on coverslips overnight, followed by interaction with apoptotic cells for 2 h, followed by washing. Cells were primed with LPS and then stained with 1μg/mL acridine orange for 15 min, and immediately stimulated for 2.5 h with DSS. Apoptotic cells were stained with 20μg/mL DAPI prior to interaction. Confocal microscopy analyses were performed with a Zeiss LSM710 instrument.

### DSS induction of colitis

Mice were treated by a single apoptotic cell infusion into tail vain containing 25-30x10^6^ cells in PBS. Colitis was then induced by oral administration of 3% (w/v) DSS solution (m.w. 36,000–50,000; MP Biomedicals) ad libitum in drinking water for 7–9 days until sacrifice. The control group received distilled water. In some *Nlrp3*-deficient mice treatment with a combination of antibiotics was investigated. Mice received drinking water with or without ampicillin (1 mg/ml), metronidazole (1 mg/ml), neomycin (1 mg/ml), and vancomycin (0.5 mg/ml).

### General assessment of colitis

Mice were sacrificed when symptoms of clinical disease became apparent in control groups, usually around 7–9 days. IBD was assessed using a standard IBD Clinical Score by daily measurements of weight change, stool consistency, and hematochezia, as described elsewhere [18], with modification. No weight loss was counted as 0, weight loss of 1 to 5% as 1, 5 to 10% as 2, 10 to 20% as 3, and >20% as 4 points. For stool consistency, 0 points were awarded for well-formed pellets, 2 for pasty and semi-formed stools that did not stick to the anus, and 4 for liquid stools that did stick to the anus. Bleeding was scored as 0 points for no blood in hemoccult, 2 for positive hemoccult, and 4 for gross bleeding. These scores were added to form a total clinical score that ranged from 0 (healthy) to 12 (maximal colitis activity). After sacrificing the animals, colons were dissected and fixated in 4% formaldehyde, and embedded in paraffin before staining with hematoxylin and eosin. Histological quantification of mucosal damage, presence and extent of inflammation, crypt damage, and percent involvement, with a range from 0 to 4, was performed on distal colon sections of the specimens. Specimens and treatment groups were blinded before histological quantification.

### Immunohistochemistry

Paraffin-embedded slides from Balb/c mice were deparaffinized and incubated in 3% H2O2. Antigen unmasking was carried out by microwave heating (20 min) in 10 mM Tris buffer containing 1 mM EDTA. Slides were incubated with primary antibodies anti-COX2, anti-MPO, anti-pNF-κB, and anti-pIκBα diluted in CAS-Block (Invitrogen), or with CAS-Block alone as a control. Appropriate secondary antibodies (Nichirei) were then added and slides were incubated at room temperature for 30 min. Color was developed using the DAB substrate kit (Thermo Scientific) followed by counterstaining with Mayer’s hematoxylin (Sigma Aldrich). Controls without addition of primary antibody showed low or no background staining in all cases.

### Statistical analysis

All data are expressed as mean ± SEM. The statistical significance of the differences was evaluated by unpaired t-test (two-tailed) or one way ANOVA with Tukey's multiple comparison tests. *P* values of 0.05 or less were considered to be statistically significant.

## Results

### DSS induces caspase-1-dependent pro-IL-1β processing via NLRP3 inflammasome activation in murine macrophages

IL-1β is a proinflammatory cytokine produced primarily by activated monocytes and macrophages, which is involved in the regulation of immune responses as well as the pathogenesis of several acute and chronic inflammatory diseases. Release of IL-1β is mediated by a two-step process: first transcriptional induction of pro-IL-1β, and then caspase-mediated cleavage for the generation and secretion of IL-1β [19]. TLR triggering is important for enhanced transcription of pro-IL-1β and pro-IL-18, and is in fact needed for the DSS effect. However the inflammasome is needed for the release of IL-1β. We were interested to examine the possible role of apoptotic cells in negative regulation of the inflammasome using both in vitro and in vivo models. Enhanced production of IL-1β has been detected upon exposure of murine macrophages to DSS [20], and more recently was shown in vitro and in vivo to be NLRP3 inflammasome-dependent [17]. Hence, we generated murine macrophages and exposed them to DSS. In agreement with the previous studies [17,20], we found that DSS induces the release of IL-1β from murine macrophages (Figure A in S1 File). Pro-IL-1β is cleaved into its active form by caspase-1, thus inhibition of caspase-1 by the specific inhibitor z-YVAD-fmk peptide led to a marked inhibition of IL-1β release (Figure A in S1 File, *p*<0.02, t test), demonstrating the role of caspase-1 in DSS-mediated IL-1β release. Activation of the NLRP3 inflammasome is K+ efflux-dependent, lysosomal-dependent, and reactive oxygen species (ROS)-dependent [21]. Indeed, blocking K+ efflux with high concentrations of KCl inhibited DSS-mediated IL-1β release (Figure A in S1 File, *p*<0.03, t test), and, similarly, blocking lysosomal acidification with bafilomycin, an inhibitor of vacuolar H+ ATPase, inhibited IL-1β secretion (Figure A in S1 File, *p*<0.04, t test). Finally, inhibition of ROS generation by N-acetyl-L-cysteine (NAC) significantly inhibited IL-1β secretion (Figure A in S1 File, *p*<0.05, t test).

**Fig 1:**
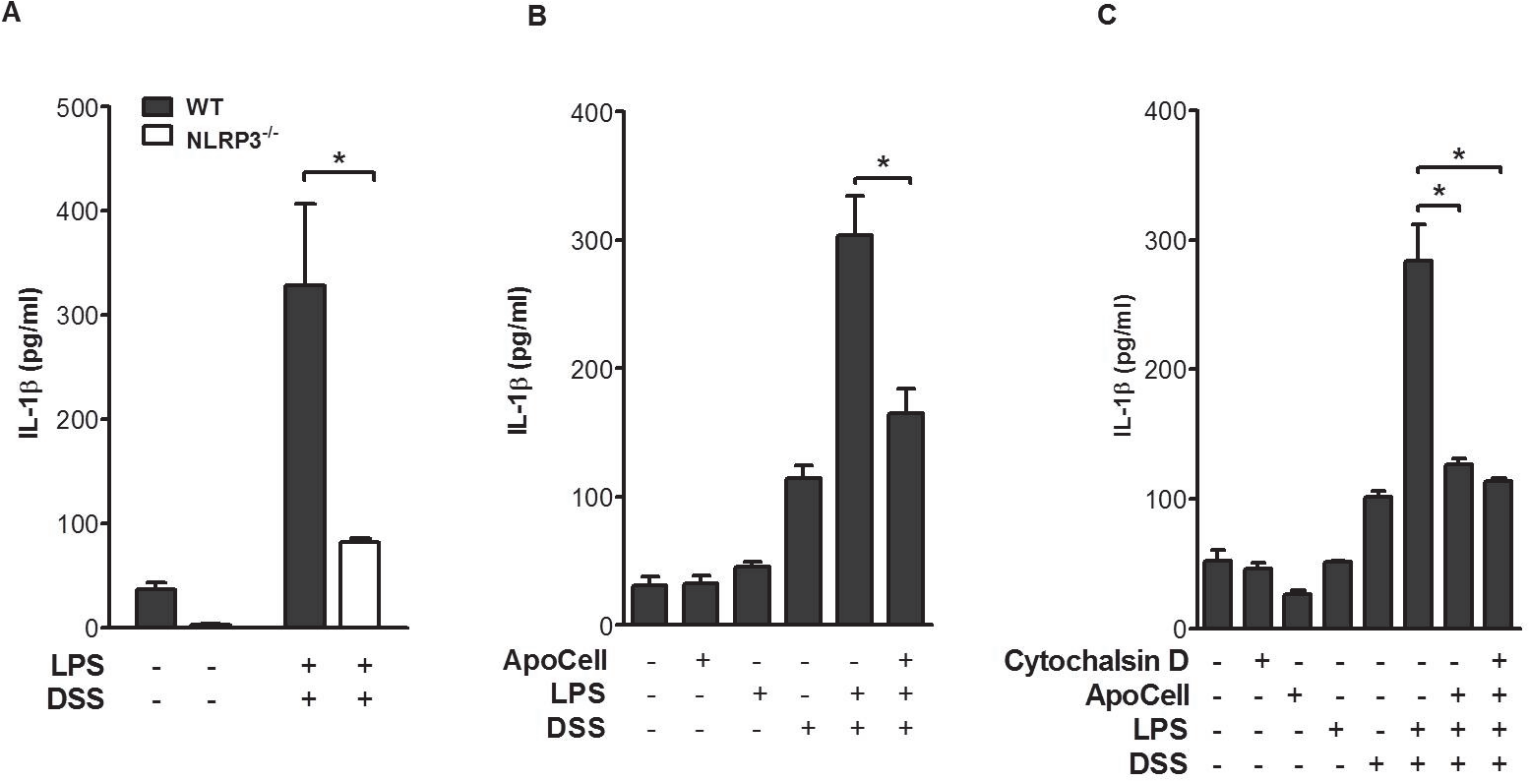
Apoptotic cells inhibit IL-1β secretion and processing in response to DSS is mediated by the NLRP3 inflammasome. **(A)** pMΦ from wild type (WT) and NLRP3-deficient mice (*Nlrp3* ^*-/-*^) and following 4 weeks of cohousing, were treated with 3% DSS in the presence of LPS priming. **(B)** Influence of apoptotic cell treatment on IL-1β release by pMΦ. Macrophages were treated with apoptotic cells (1:8) prior to LPS and 3% DSS treatment. IL-1β was determined in the supernatant by ELISA. Shown are representative data as means ± SEM of 3-to-5 independent experiments done in triplicate (*p<0.01 t-test). **(C)** The inhibition effect is a direct consequence of apoptotic cell recognition, independent of engulfment. Macrophages were incubated without or with 2 μM cytochalasin D for 45 min before the addition of apoptotic cells and DSS challenge. Shown are representative data as means ± SEM of 2 independent experiments done in triplicate (*p<0.01, one way ANOVA).

Despite these convincing data, conflicting observations suggested opposite dependency in DSS colitis on the NLRP3 inflammasome [17,22–24]. These conflicting observations probably failed to consider recent observations regarding microbiota and their effect on NLRP6 [14]. Therefore, first, we used wild type and *Nlrp3*-deficient mice bred in our facilities (A generous gift from the late J Tschopp) following cohousing of the wild type and *Nlrp3*-deficient mice [14] and exposed them to DSS in vivo. Surprisingly at first, NLRP3-deficent mice indeed showed a more severe disease suggesting a general protecting effect of NLRP3 as suggested by several groups (Figure B in S1 File). Of note, this aggravation was abolished when using antibiotics; ampicillin (1 mg/ml), metronidazole (1 mg/ml), neomycin (1 mg/ml), and vancomycin (0.5 mg/ml), suggesting the contribution of the microbiota to the colitis development. It should be also emphasize that in vivo, when using bone marrow chimeras the DSS-induced colitis protective effect was shown to be due NLRP3 signaling in non-hematopoietic cells [22]. Therefore and since we investigated resolution of inflammation in the hematopoietic system, we further examined hematopoietic cells in vitro, i.e., macrophages from NLRP3-deficient mice and exposed them to DSS. Again, in order to further neutralize the microbiota effect, we initiated experiments following cohousing of the wild type and NLRP3-deficient mice in order to allow observation of pure inflammasome function without microflora modification. Indeed, following cohousing, DSS-induced IL-1β secretion is significantly reduced in NLRP3-deficient mice (Fig 1, *p*<0.02, t test).

Taken together, these results suggest that DSS-mediated IL-1β secretion is caspase-1 and NLRP3 activation-dependent in macrophages.

### Apoptotic cells inhibit inflammasome-induced IL-1β release from macrophages

Other labs [5] and our group [9] have shown inhibition of IL-1β release by macrophages exposed to apoptotic cells and then to TLR agonists, but it is not known whether they can inhibit secretion upon NLRP3-specific activation. Macrophages were exposed to lipopolysaccharide (LPS) and DSS with or without earlier interaction with apoptotic cells for two hours. Prior apoptotic cell treatment significantly inhibited IL-1β secretion from macrophages exposed to DSS, with similar inhibition of z-YVAD, KCl, bafilomycin and NAC (Fig 1, *p*<0.02, t test), suggesting that apoptotic cells negatively signal the inflammasome pathway. Apoptotic cells were shown to extract their anti-inflammatory effect at the recognition level by macrophages [25]. To further illustrate the level of apoptotic cells negative signaling we have used cytochalasin D, a pharmacologic agent that inhibits actin polymerization, to prevent and eliminate engulfment. Using this approach we were able to show that binding of apoptotic cells to macrophages without engulfment is fully sufficient for inhibition of IL-1β secretion (Fig 1, *p<0.01, one way ANOVA).

**Fig 2:**
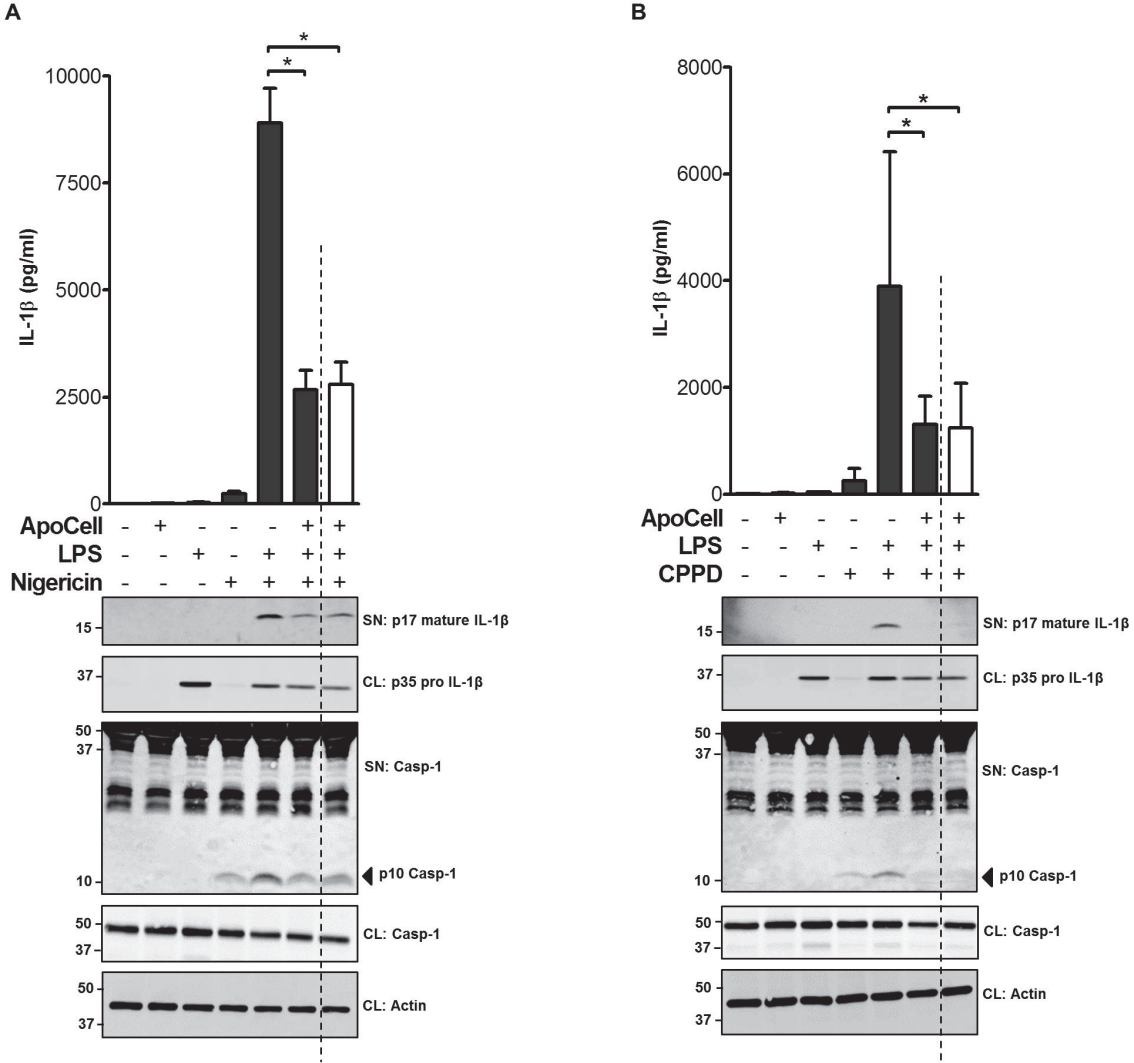
IL-1β inhibition by apoptotic cells pre and post NF-κB triggering by LPS. (A-B). IL-1β measured by ELISA (**Upper panel**) and western blot (**Lower panel**). IL-1β and caspase-1 were measured in supernatant (SN) and/or intracellular in cell lysate (CL). B6 pMΦ cells were incubated either in the presence of apoptotic cells for 2h followed by LPS priming for 1h (sixth from left, dark bar), or first primed with LPS (to promote NF-κB signaling) for 1h and then incubated with apoptotic cells for 2h (seventh from left, white bar, separated by dashed line). pMΦ were then incubated with various inflammasome inducers. **A**: nigericin 2.5μM; **B**: calcium pyrophosphate dihydrate 200μg/mL (CPPD). An anti-mouse actin served as a loading control. Shown are data for A-B, as means ± SEM of 3 independent experiments done in duplicates and WB representative data of three experiments (*p<0.001, one way ANOVA).

Given the need for TLR triggering through NF-κB signaling, and the fact that apoptotic cells can inhibit NF-κB, and therefore inhibit IL-1β secretion in the absence of inflammasome inhibition, we initiated a set of experiments to elucidate whether IL-1β secretion is inhibited both at NF-κB and NLRP3 levels by apoptotic cells. Resident peritoneal macrophages (pMΦ) were either incubated with apoptotic cells, washed, and primed with LPS following stimulation with various inflammasome inducers, or were first primed with LPS, allowing accumulation of de novo pro-IL-1β transcription and then treated with apoptotic cells and inducers. Using this approach, we were able to distinguish between pre- and post-transcription inhibitions. Indeed as expected, apoptotic cells treatment inhibited the secretion of activated IL-1β at pre-transcription levels attributing it to NF-κB pathway inhibition. More importantly, the inhibition effect e.g. IL-1β secretion, was also observed after the accumulation of de novo IL-1β that is, after LPS priming. This inhibition effect was obtained using two different activators of NLRP3 triggering mechanisms; including nigericin and calcium pirophosphate (CPPD), suggesting a more direct inhibitory effect on NLRP3 inflammasome (Fig 2, *p*<0.001, one way ANOVA). The results were further verified by western blot analysis showing a diminished cleaved IL-1β subunit in the supernatant of macrophages treated with LPS prior to apoptotic cell treatment (Fig 2, lower panel). Of note, LPS priming by itself, leads to accumulation of de novo pro-IL-1β in macrophages as can be seen in the cell lysate fraction (CL) but none in the supernatant (SN) (third lane from left in Fig 2 lower panel (WB)). The reduction in IL-1β levels was seen even if NF-κB triggering with LPS was allowed before exposure to apoptotic cells. To exclude an alternative IL-1β secretion mechanism, we have verified IL-1β secretion in pMΦ from *Nlrp3*-deficient mice following the above treatments. Only negligible levels of IL-1β were recorded indicating a major NLRP dependent mechanism (Figure C in S1 File, *p*<0.001, one way ANOVA). Similar results are shown with caspase-1 where less activation of caspase-1 was measured at pre- and post- transcription levels following apoptotic cells treatment in the presence of different inducers. We have also verified that apoptotic cells and LPS by themselves do not affect the secretion of mature IL-1β or caspase-1 activation in the absence of inflammasome triggering. Indeed no secretion of mature IL-1β or caspase-1 activation was observed, both at the pre- and post-transcription levels, indicating the involvement of NLRP3-inflammasome (Figure D in S1 File). Taken together, apoptotic cells appear to have a distinct inhibition effects on NF-κB and NLRP3.

### Apoptotic cell treatment has a dramatic clinical effect on DSS inflammatory colitis severity

**Fig 3:**
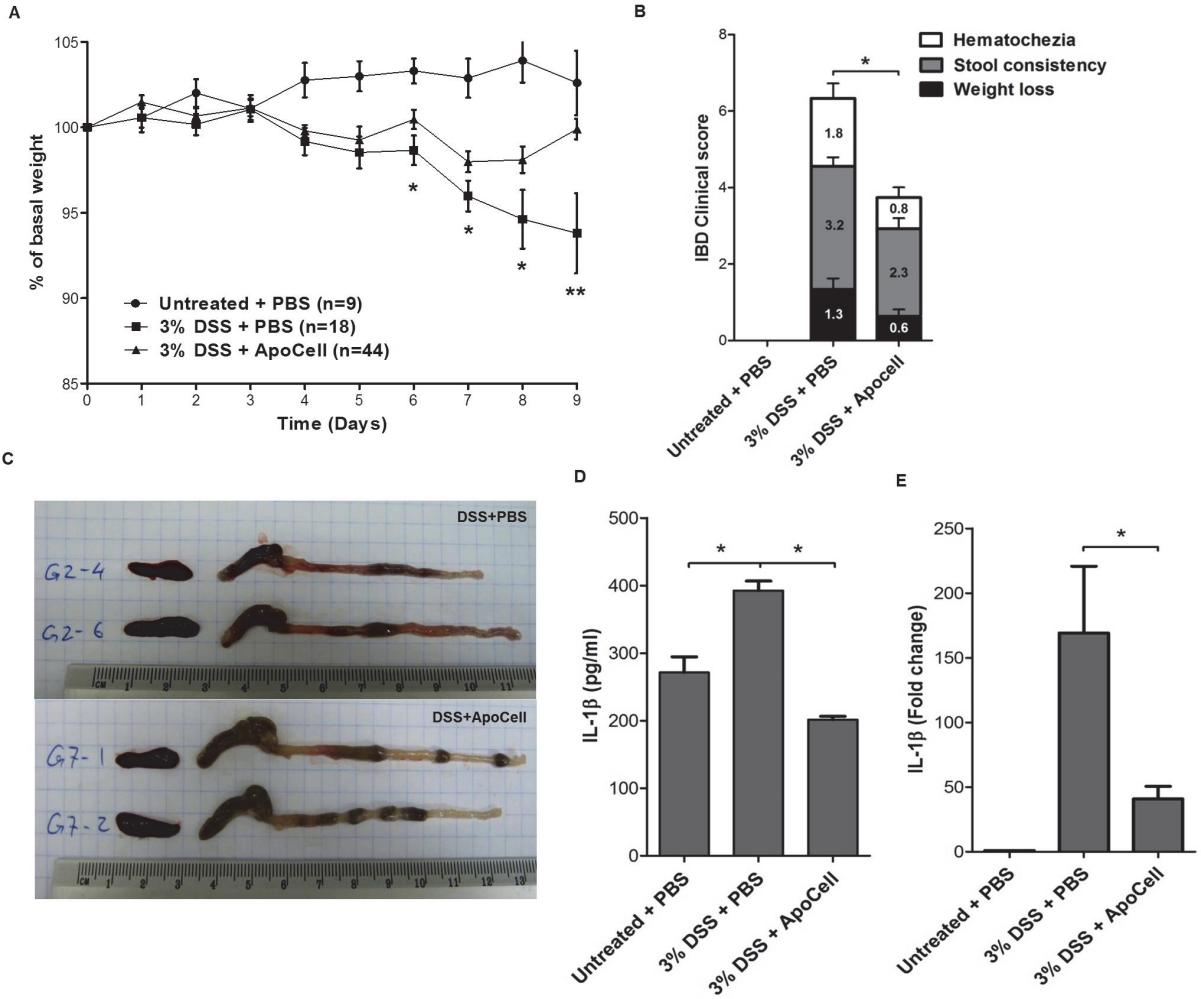
Apoptotic cell treatment protects mice from DSS-induced colitis. Balb/c mice were offered distilled water (filled circles), or distilled water with 3% DSS orally ad libitum with treatment of PBS (filled squares) or apoptotic cell (filled triangles). (**A**) Mean weight of indicated animal number per group (*p<0.05, **p<0.001, t-test). (**B**) IBD Clinical Score. Numbers inside boxes indicate the mean score of each parameter with error bar (*p<0.001, t-test). Data is presented as mean ± SEM of 3 independent experiments. Weight change, hematochezia and stool consistency were monitored daily. (**C**) Macroscopic changes of colon and spleen in DSS-treated mice. Photographs of the dissected large intestines and spleens of four mice treated with 3% DSS without- (DSS+PBS) or with apoptotic cell treatment (DSS+ApoCell). **(D)** IL-1β cytokine level in colonic homogenate from DSS-treated mice. Levels of IL-1β were analyzed by ELISA. Data is presented as mean ± SEM, 3 mice per group (*p<0.001, one way ANOVA). **(E)** IL-1β mRNA levels in colonic homogenate from DSS-treated mice. mRNA was measured by RT-PCR and normalized to untreated colons. Data is presented as mean ± SEM, 4–5 mice per group (*p<0.02, t-test)

Next we were interested in examining this anti-inflammasome effect as a possible therapeutic treatment of a single infusion of apoptotic cells on a "danger" inducing agent; hence we evaluated the effect of apoptotic cell treatment in DSS-mediated intestinal inflammation, in vivo. Mice received 3% DSS in their drinking water for a period of 7–9 days. IBD clinical score parameters, including body weight, the presence of latent or gross blood per rectum, and stool consistency [18], were determined daily. Mice treated with apoptotic cells in addition to DSS showed significant less body weight loss starting from day 6, compared to mice treated only with DSS (Fig 3, *p*<0.05 and 0.001, t test). Clinical score analysis revealed significantly less severe colitis in mice treated with apoptotic cells, in all parameters evaluated (Fig 3, *p*<0.01, t test). On macroscopic examination, DSS-treated colons were severely inflamed and hyperemic, and they contained fewer feces due to massive diarrhea. When treated with apoptotic cells, colon were less effected and were longer than DSS only treated colons (9.4 ±0.14cm vs. 8.9±0.2, *p*<0.05, t test) (Fig 3). We next measured the levels of IL-1β in colonic homogenates of mice treated with apoptotic cells followed by DSS in vivo. After 7 days of DSS intake, IL-1β concentrations were indeed significantly elevated, But in mice treated with only a single apoptotic cell injection prior to DSS intake, a significant decrease in IL-1β was measured (Fig 3, *p*<0.001, one way ANOVA). In addition, Gut *IL-1B* RNA expression levels were lower following apoptotic cell treatment as detected in real-time PCR (Fig 3, *p*<0.02, t-test). Interestingly and in support of our hypothesis, aggravation of DSS-colitis in *nlrp3*-deficient mice was not ameliorated by apoptotic cells treatment (Figure B in S1 File).

**Fig 4:**
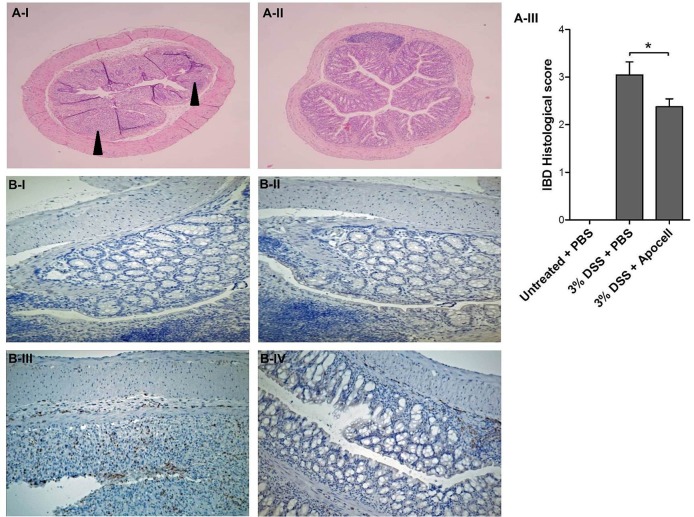
Histological appearance and neutrophil infiltration of distal colon sections. (**A**) H&E appearance. (**A-I**) DSS uptake leads to severe epithelial damage (black arrowed) while (**A-II**) apoptotic cell treatment maintain integrity of treated mice (**A-III**) histological score of distal colon sections of DSS-treated Balb/c mice Results from 3 independent experiments (*p<0.05, t-test). (**B**) Apoptotic cell treatment inhibits neutrophil accumulation in inflamed colon. Mouse colon tissue sections were stained by immunohistochemistry assay using a rabbit monoclonal antibody against mouse myeloperoxidase (MPO). After immunostaining, slides were counterstained by hematoxylin. Images show the MPO stain followed by HRP-anti rabbit secondary antibody. All images are x200. **(B-I)** Staining control. Untreated colon stained with HRP-anti rabbit secondary antibody only, without anti-MPO. **(B-II)** Normal colon control. MPO-stained neutrophils in untreated colon. **(B-III)** DSS treatment. MPO-stained neutrophils in 3% DSS treated colon (3% DSS+PBS). **(B-IV)** Apoptotic cell & DSS treatment. MPO-stained neutrophils in 3% DSS-treated colon with apoptotic cell infusion (3% DSS+ApoCell).

Apoptotic cell treatment also had a histological anti-inflammatory effect on DSS and colitis severity. Biopsies showed significantly less severe mucosal infiltration by inflammatory cells and reduced tissue damage, with a significantly improved histological colitis severity score performed by a blinded pathologist (Fig 4, *p*<0.05, t test).

We further evaluated the range of neutrophils by myeloperoxidase (MPO) staining and colon inflammation by Cyclooxygenase 2 (Cox2) staining. Neutrophil infiltration was markedly higher in colon tissue of mice who had not received apoptotic cell treatment as shown both in hematoxylin and MPO staining (Fig 4). Cox2 immunostaining showed a dramatic elevation in the number of positive cells in DSS-treated colons compared to non-treated colons (Figure E in S1 File). When apoptotic cell treatment was applied, a marked reduction was observed.

**Fig 5:**
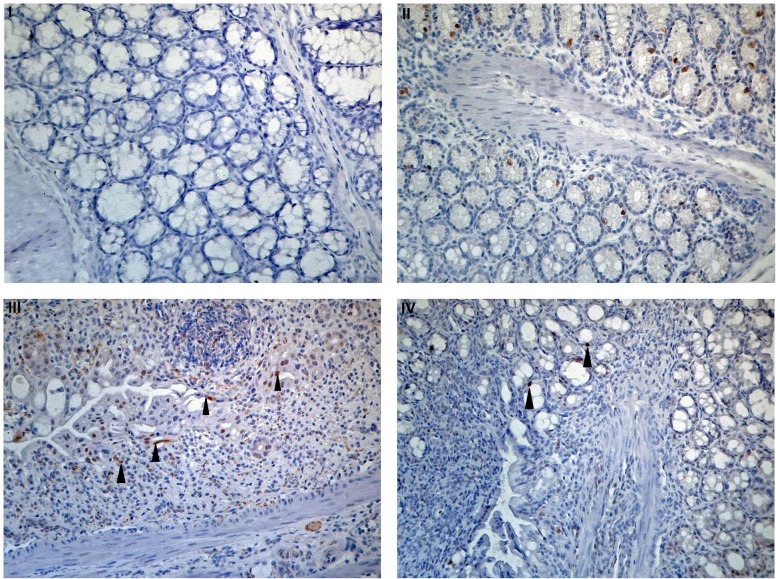
Apoptotic cell treatment inhibits NF-κB in DSS-induced colitis. Mouse colon tissue sections were stained by immunohistochemistry assay using an antibody against mouse phospho-NF-κB (pNF-κB) p65. After immunostaining, slides were counterstained by hematoxylin. Images show pNF-κB p65 staining. All images are x200. **(I)** Untreated colon stained with HRP-anti rabbit secondary antibody only, without anti-NF-κB. **(II)** pNF-κB p65 staining in untreated colon. **(III)** Large pNF-κB p65 positive stain (black arrow) in 3% DSS-treated colon (3% DSS+PBS). **(IV)** Fewer pNF-κB p65 positive stain (black arrow) in 3% DSS treated colon with apoptotic cell infusion (3% DSS+ApoCell).

Additionally, we have analyzed the phosphorylation of p65 NF-κB and IκBα in colonic tissue. An appreciably higher number of pNF-κB-positive cells were observed in colon treated solely with DSS compared with colon that was also treated with apoptotic cells (Fig 5). Inhibition of NF-κB signaling was further confirmed by the upstream inhibitory protein IκBα where reduced number of cells that were positive for pIκBα when treated with apoptotic cells, as detected by immunostaining (Figure F in S1 File).

### The apoptotic cell anti-inflammasome effect is mediated via ROS, lysosome stabilization, and K^+^ efflux

**Fig 6:**
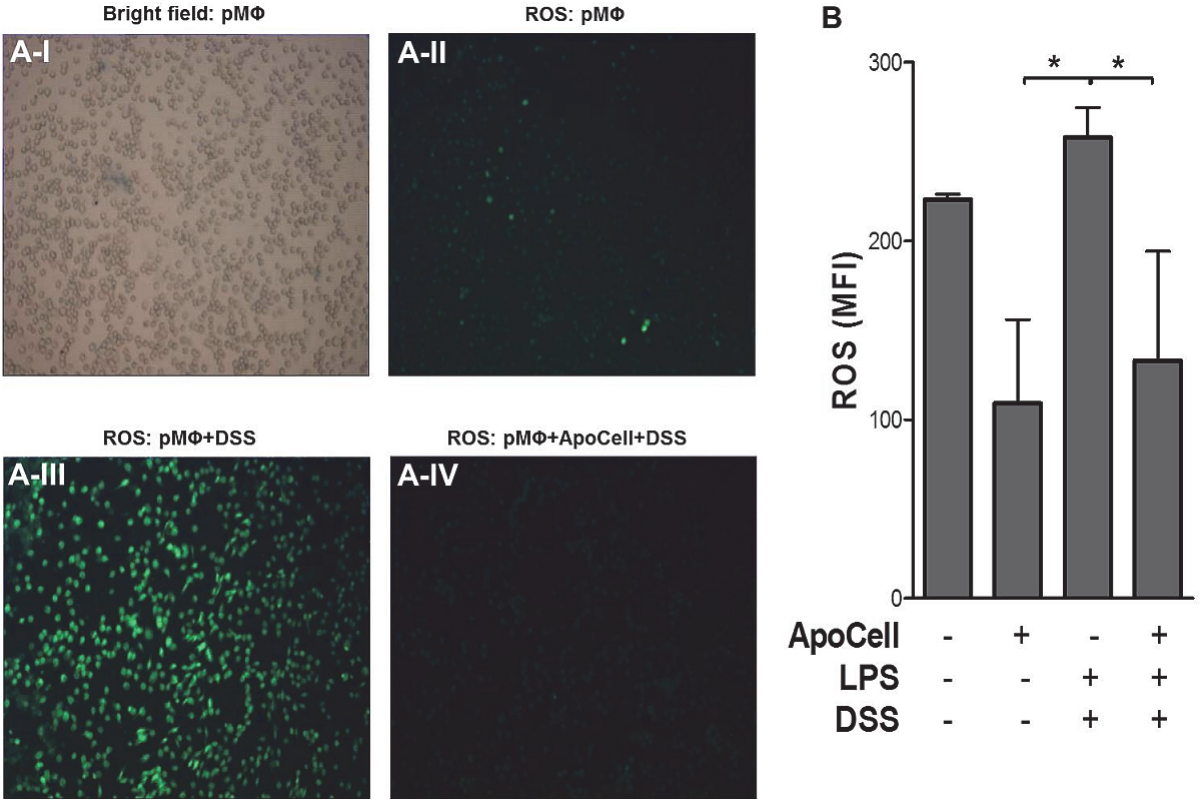
Reactive oxygen species (ROS) in pMΦ. **(A)** Upper Panel: **I**. pMΦ as seen in bright field. **II**. pMΦ seen by fluorescent microscope following incubation with an ROS-sensitive dye (1μM). **III**. pMΦ following 3% DSS treatment. Generation of ROS is seen. **IV**. pMΦ following 3% DSS and apoptotic cell treatment. Inhibition of ROS generation is seen. Original magnification: All panels x100. pMΦ extracted from mice were seeded overnight onto eight-chamber slides at a density of 1x10^5^ cells/chamber. After washing, pMΦ were treated for 2h with apoptotic cells followed by treatment with 3% DSS for 30 min. Negative control samples were treated with media only. ROS generation was determined by fluorescence microscopy using a fluorescein fluorescent probe for 30 min with a green filter. The experiments were repeated 3 times, independently; one representative experiment is shown. **(B)** Flow-cytometer analysis of pMΦ stained with ROS-sensitive dye. ROS generation was determined by flow-cytometer using a fluorescence probe as above, excluding dead cells base on FSC/SSC parameters. Shown are means ± SEM of 3 experiments done in triplicates (*p<0.05, one way ANOVA).

Three main mechanisms leading to NLRP3 activation has been suggested [21]. Activation of the NLRP3 inflammasome was suggested to be ROS-dependent, and indeed many NLRP3 stimulators also induce ROS generation [26,27]. DSS was also found to generate ROS during NLRP3 activation and accumulation of IL-1β [17,28]. We were interested in examining whether apoptotic cell treatment has an effect on ROS generation. In agreement with the previous observations [17,28], peritoneal macrophages incubated with DSS were found to induce ROS, as shown in Fig 6, where ROS production was assessed with both fluorescent microscopy and real time flow-cytometer. When macrophages were pretreated with apoptotic cells and then treated with DSS, a marked and significant reduction in ROS generation was seen (Fig 6, *p*<0.05, one way ANOVA).

**Fig 7:**
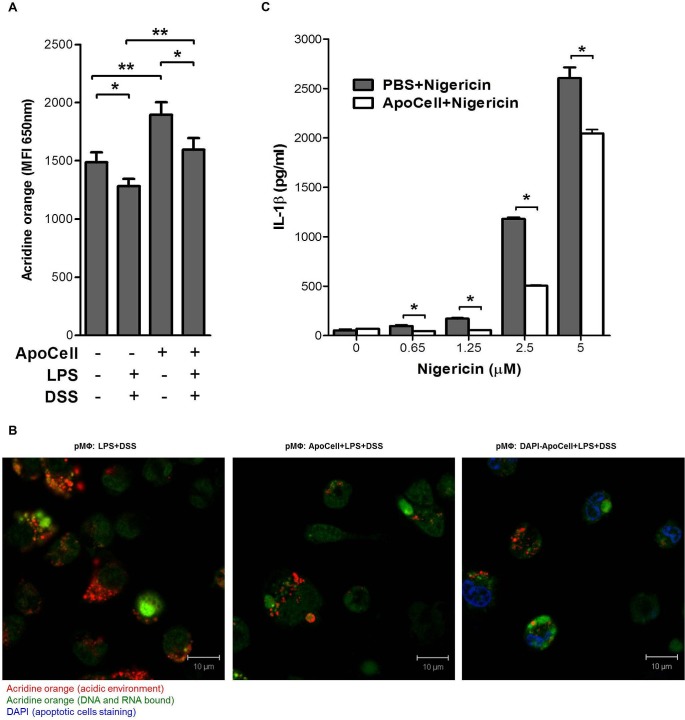
Lysosomal damage and K+ efflux in pMΦ. **(A)** Flow-cytometer analysis of B6 pMΦ treated for 2h with apoptotic cells and/or 24h with DSS were stained with fluorochrome acridine orange (AO). Loss of fluorescence, which correlates with reduced numbers of lysosomes, was analyzed by flow-cytometer, excluding dead cells base on FSC/SSC parameters. Shown are means ± SEM of 4 independent experiments (*p<0.05, **p<0.03, one way ANOVA). **(B)** confocal microscopy of LPS primed pMΦ incubated (or not; left) for 2h with apoptotic cells (middle) or DAPI-stained apoptotic cells (right), stained with 1μg/mL acridine orange for 15 min and then incubated for 2.5 h with 3% DSS. Representative data from four experiments. **(C)** Apoptotic cell treatment inhibits nigericin-induced IL-1β secretion. B6 pMΦ cells were treated with nigericin at the indicated concentrations in the presence of LPS priming, with or without apoptotic cell treatment (*p<0.01, unpaired t-test).

The second mechanism described as an important in NLRP3 activation is lysosomal damage, leading to cytosolic release of lysosomal content that triggers the inflammasome [21]. Bauer et al. also suggested that DSS triggers inflammasomes by lysosomal damage. To test whether apoptotic cells prevent lysosomal damage, we used cytosolic staining with acridine orange, a dye with green fluorescence when monomericly bonded to DNA and RNA and red fluorescence with dimerization in acidic compartments. The extent of the red fluorescence correlates with the level of intracellular acidic lysosomes. DSS treatment resulted in a significant decrease in red fluorescence intensity (Fig 7, *p*<0.05, one way ANOVA), indicating lysosomal damage, and in agreement with previous findings for DSS [17] and crystals [29]. Indeed when we treated our macrophages with apoptotic cells prior to the DSS challenge, a significant increase in the number of acidic compartments was detected suggesting stabilization of the lysosomal compartment (Fig 7). This observation was confirmed using confocal microscopy, which showed a more diffuse cytosolic staining pattern when macrophages were treated with DSS, indicating rupture of lysosomes (Fig 7). However, when treated with apoptotic cells prior to DSS challenge, the lysosomes appeared intact (Fig 7). Taken together, this data suggests that lysosomal compartment stabilization is involved in inflammasome regulation by apoptotic cells.

The third NLRP3 activation mechanism was suggested to involve changes in the intracellular ionic milieu [30], either via ATP and the P2X7 receptor or by pore forming toxins [31], and possibly also involving pannexin-1 [32]. Blocking K+ efflux or applying high concentrations of K+ prevented NLRP3 inflammasome activation by many agents [27,30], including DSS [17] (Figure A in S1 File). To evaluate to role of K+ efflux in the presence of apoptotic cells, we tested whether macrophages pretreated with apoptotic cells can inhibit IL-1β secretion following LPS and nigericin challenge. IL-1β secretion was indeed inhibited up to 70% by apoptotic cells in a dose-dependent manner, although less competent at high concentrations (Fig 7).

## Discussion

Here we show, for the first time, that apoptotic cells negatively regulate the NLRP3 inflammasome and are able to downregulate the proinflammatory response induced via NLRP3 inflammasome in hemtopoeitic cells, both in vitro and in vivo. These observations explain previous observations, where apoptotic cell treatment have been shown to induce peripheral tolerance in an immunosuppressive manner [1,5,6,33–35] indicating that accumulation of apoptotic cells at an inflammatory site may be the key mechanism for termination of the inflammatory response, as seen in the amelioration of DSS-induced colitis. It has been shown that viral PYD proteins and various bacterial virulence factors that inhibit caspase-1 activation (for a review see [36]) exist as a means of anti-host attack, but our observation is that they more likely represent a homeostatic mechanism for the termination of inflammation. Although we have shown this for inflammation presumed to be sterile, it is most likely that this mechanism also stands in infectious situations.

The inflammasome triggering is a two-hit model requiring both TLR and inflammasome triggering [21]. Indeed, apoptotic cells were shown to inhibit TLRs and the NF-κB pathway [7–9]. TLR triggering is important for enhanced transcription of pro-IL-1β and pro-IL-18, and is in fact needed for the DSS effect. In this study we were able to show that apoptotic cells inhibited the secretion of activated IL-1β at both pre- and post-transcription levels and had distinct inhibition effects on NF-κB and NLRP3.

The present study may indicate involvement of three mechanisms in the resolution by apoptotic cells of inflammasome-induced inflammation. First, other groups [33,34] and our lab have shown that apoptotic cells are able to reduce and inhibit the formation of ROS, at rates similar to those shown for the chemical inhibitor NAC. It is well established that macrophages make use of toxic ROS to control microbial pathogens as part of the innate immune response and ROS were identified as major mediators of inflammatory signals believed to play a role in the development of IBD. Furthermore, generation of ROS was found to induce IL-1β via ERK phosphorylation [37]. In addition, IL-1β signals may induce ROS generation [38]. While it has been shown that DSS induces formation of ROS [17,28], we were able to see a marked reduction in ROS generation, and consequently less IL-1β secretion when macrophages were pretreated with apoptotic cells.

The second mechanism involves the lysosome. It was shown that lysosomal damage or leakage may serve as an endogenous danger signal and is sensed by the NLRP3 inflammasome [17,29]. We have analyzed involvement of the lysosome vacuole, and although clearance of apoptotic cell is mediated via the phagolysosomal pathway [39], it seems that other mechanism are involved since recognition of apoptotic is sufficient to extract tolerance. We found that lysosomes from peritoneal macrophages that were introduce to apoptotic cells were more stable to DSS challenge, and were not affected or damaged. This may point to the notion that apoptotic cells are multifactorial and may desensitize lysosome for at least 24 h after apoptotic recognition. Taken together, these results demonstrate a mechanism of inflammasome inhibition and resolution of inflammation stemming from apoptotic cell clearance.

Inflammasomes were also suggested to be activated in response to signaling pathways that deplete intracellular potassium, such as the potassium ionophore nigericin [31]. When macrophages were pretreated with apoptotic cells, nigericin-induced IL-1β secretion was significantly inhibited. The means by which apoptotic cells inhibit nigericin-induced IL-1β secretion in not clear and only partially can be explained by a direct inflammasome upstream inhibitory mechanism that is perhaps best mediated via NF-κB signaling. This observation illustrates a mechanism of regulation of inflammation that could take place in both infectious and non-infectious inflammatory conditions. Additional related possible mechanisms include functional sub-group of NLRs that negatively regulate inflammation [40], including the possible effect of apoptotic cells on NLRP12, a suppressor of pro-inflammatory cytokine and chemokine production downstream of TLRs by targeting multiple points in the NF-κB pathway. However it is clear that failure to clear apoptotic cells will trigger persistence inflammasome-dependent inflammation as perhaps is seen in failure to clear intracellular organelles [41].

The in vitro and in vivo properties of apoptotic cells suggest a potential use in a broad range of inflammatory and immune-mediated conditions, such as graft versus host disease [42] and IBD. In the present study a single infusion of apoptotic cells significantly ameliorated both the clinical score and histological appearance of a DSS-induced colitis. In summary, apoptotic cells infusion is beneficial in mice models of IBD and inhibits both inflammasome- and NF-κB-dependent inflammation.

## Supporting Information

S1 File Supporting figures. Figure A in S1 File. Dextran sodium sulfate (DSS) induces caspase-1-mediated IL-1β release from murine macrophages. Figure B in S1 File. Apoptotic cell treatment in *nlrp*3 deficient mice. Figure C in S1 File. Negligible IL-1β secretion in *nlrp*3 deficient mice. Figure D in S1 File. Influence of apoptotic cells and LPS on IL-1β and caspase-1 activation. Figure E in S1 File. Apoptotic cell treatment inhibits cyclooxygenase-2 (COX-2) in DSS-induced colitis. Figure F in S1 File. Apoptotic cell treatment inhibits Iκ-Bα phosphorylation in DSS-induced colitis.
(DOCX)
