# Supplementary material for: Apoptotic Cells Induce NF-κB and Inflammasome Negative Signaling
Source: PLoS One. 2015 Mar 30;10(3):e0122440. doi: 10.1371/journal.pone.0122440 (PMC4379019; doi:10.1371/journal.pone.0122440)
Supplement: S1 File — Figure A in S1 File. Dextran sodium sulfate (DSS) induces caspase-1-mediated IL-1β release from murine macrophages. Figure B in S1 File. Apoptotic cell treatment in nlrp3 deficient mice. Figure C in S1 File. Negligible IL-1β secretion in nlrp3 deficient mice. Figure D in S1 File. Influence of apoptotic cells and LPS on IL-1β and caspase-1 activation. Figure E in S1 File. Apoptotic cell treatment inhibits cyclooxygenase-2 (COX-2) in DSS-induced colitis. Figure F in S1 File. Apoptotic cell treatment inhibits Iκ-Bα phosphorylation in DSS-induced colitis. (DOCX) [file pone.0122440.s001.docx]

**Supplementary figures**


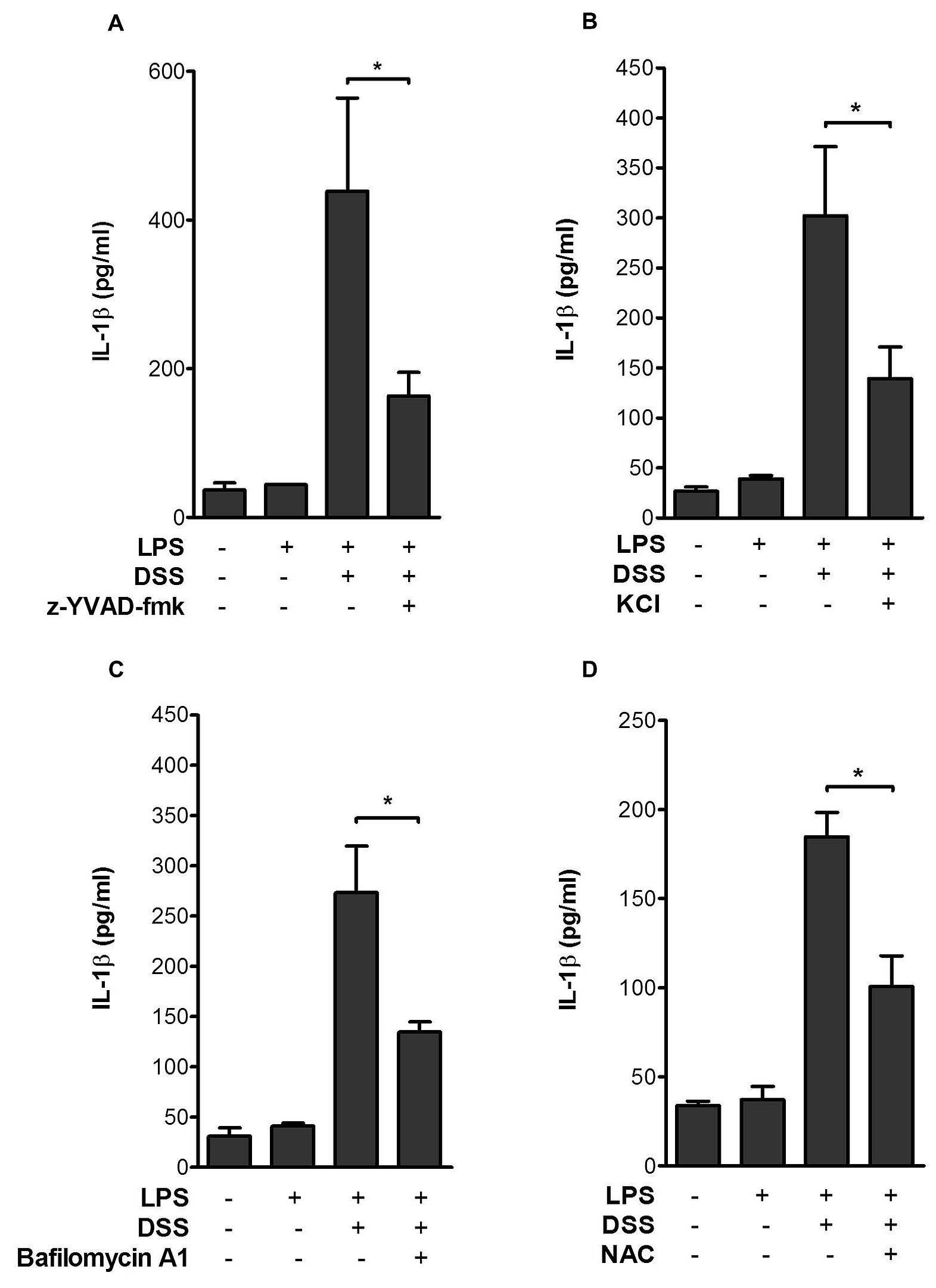


**Figure A.** **Dextran sodium sulfate (DSS) induces caspase-1-mediated IL-1β release from murine macrophages.** (**A**) Influence of the caspase-1 inhibitor z-YVAD-fmk on IL-1β release by pMΦ. LPS-primed pMΦ were treated with z-YVAD-fmk (10 µM), followed by 3% DSS. (**B**) Inflammasome activation is dependent on K+ efflux, with high K+ (130mM) concentrations blocking secretion of IL-1β. (**C**) Activation of the NLRP3 inflammasome by DSS requires lysosomal maturation and reactive oxygen species (ROS). pMΦ incubated with bafilomycin A1 (10 nM), or (**D**) with the ROS inhibitor N-acetyl-L-cysteine (NAC) (20mM) before treatment with 3% DSS. IL-1β was determined in the supernatant by ELISA. Shown are representative data as means ± SEM of 3-to-5 independent experiments done in triplicate (**p*<0.05, t test).

**
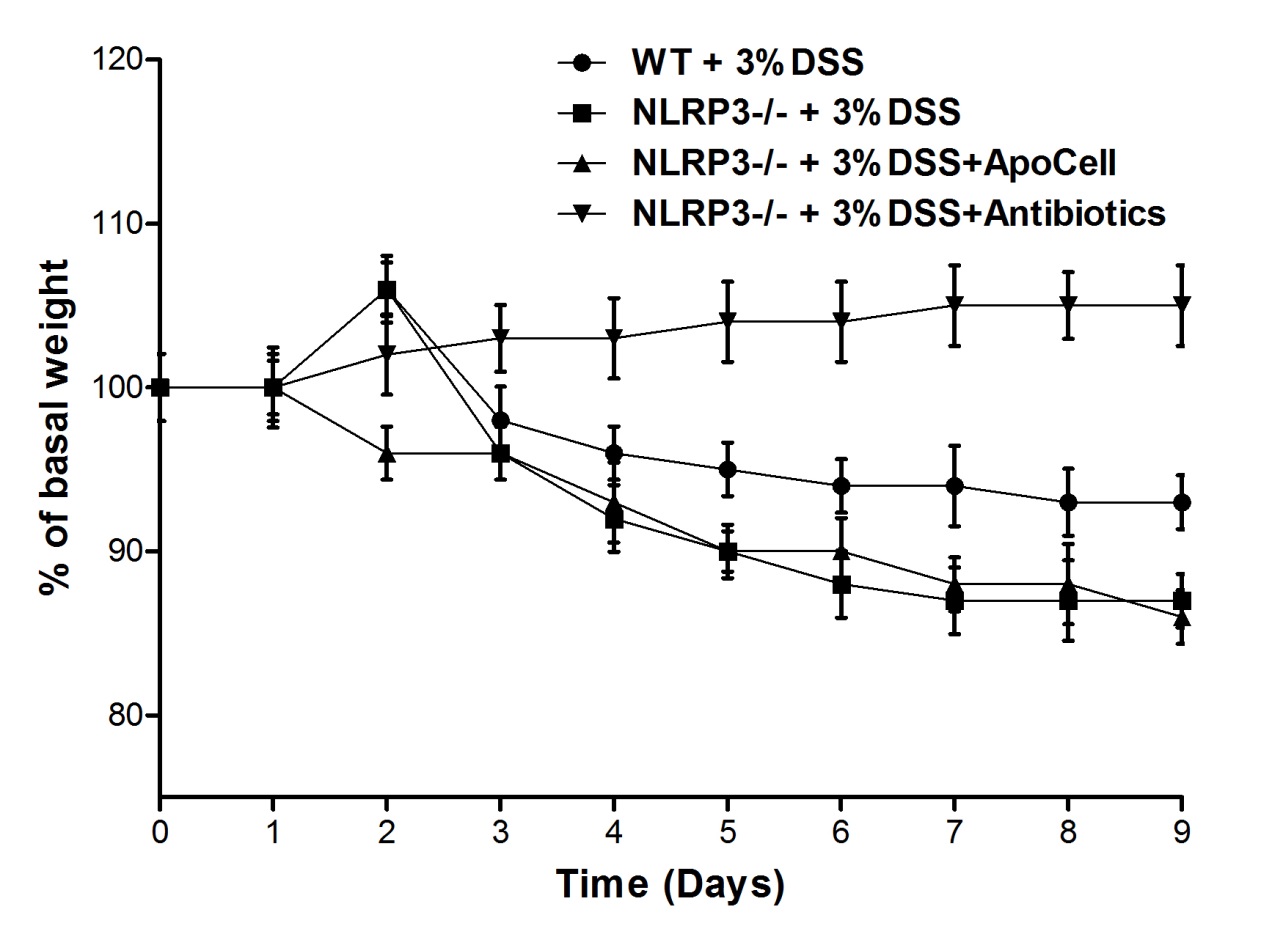
**

**Figure B. Apoptotic cell treatment in *nlrp*3 deficient mice.** Apoptotic cell treatment was not associated with amelioration of DSS-colitis in *nlrp*3-deficient mice. Treatment with antibiotics protects *nlrp*3-deficient mice from DSS-induced colitis. Mice were offered distilled water with 3% DSS orally ad libitum with treatment or antibiotics in one group (filled triangles). Mean weight of 6 animal number per group (p<0.0003, one way ANOVA).

**
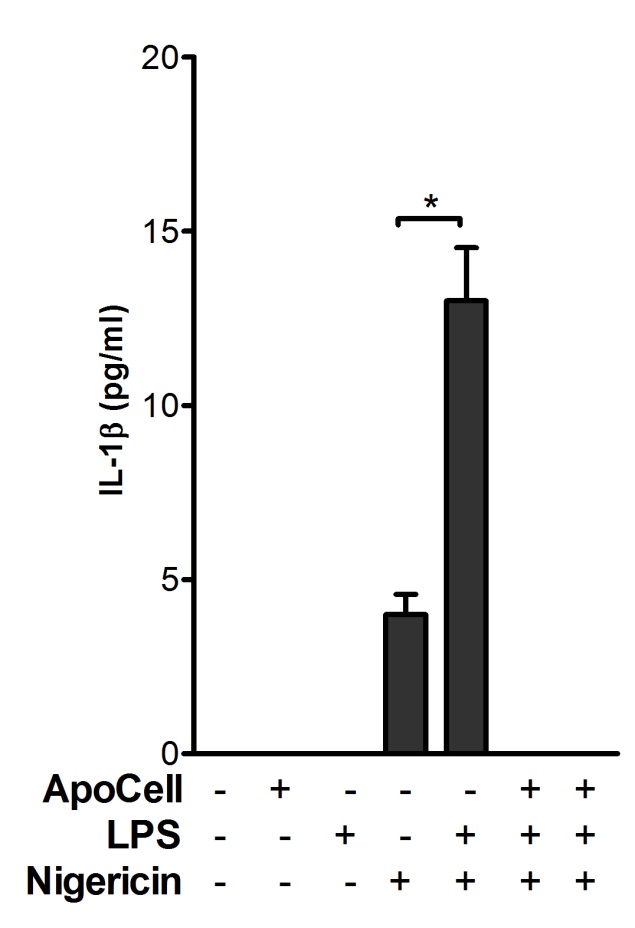
**

**Figure C. Negligible IL-1β secretion in nlrp3 deficient mice.** IL-1β measured by ELISA. B6 pMΦ cells were incubated either in the presence of apoptotic cells for 2h followed by LPS priming for 1h (sixth from left, dark bar), or first primed with LPS (to promote NF-κB signaling) for 1h and then incubated with apoptotic cells for 2h. pMΦ were then incubated with inflammasome inducer nigericin (2.5µM). Shown are data as means ± SEM of 3 independent experiments done in duplicates (**p*<0.001, one way ANOVA).

**
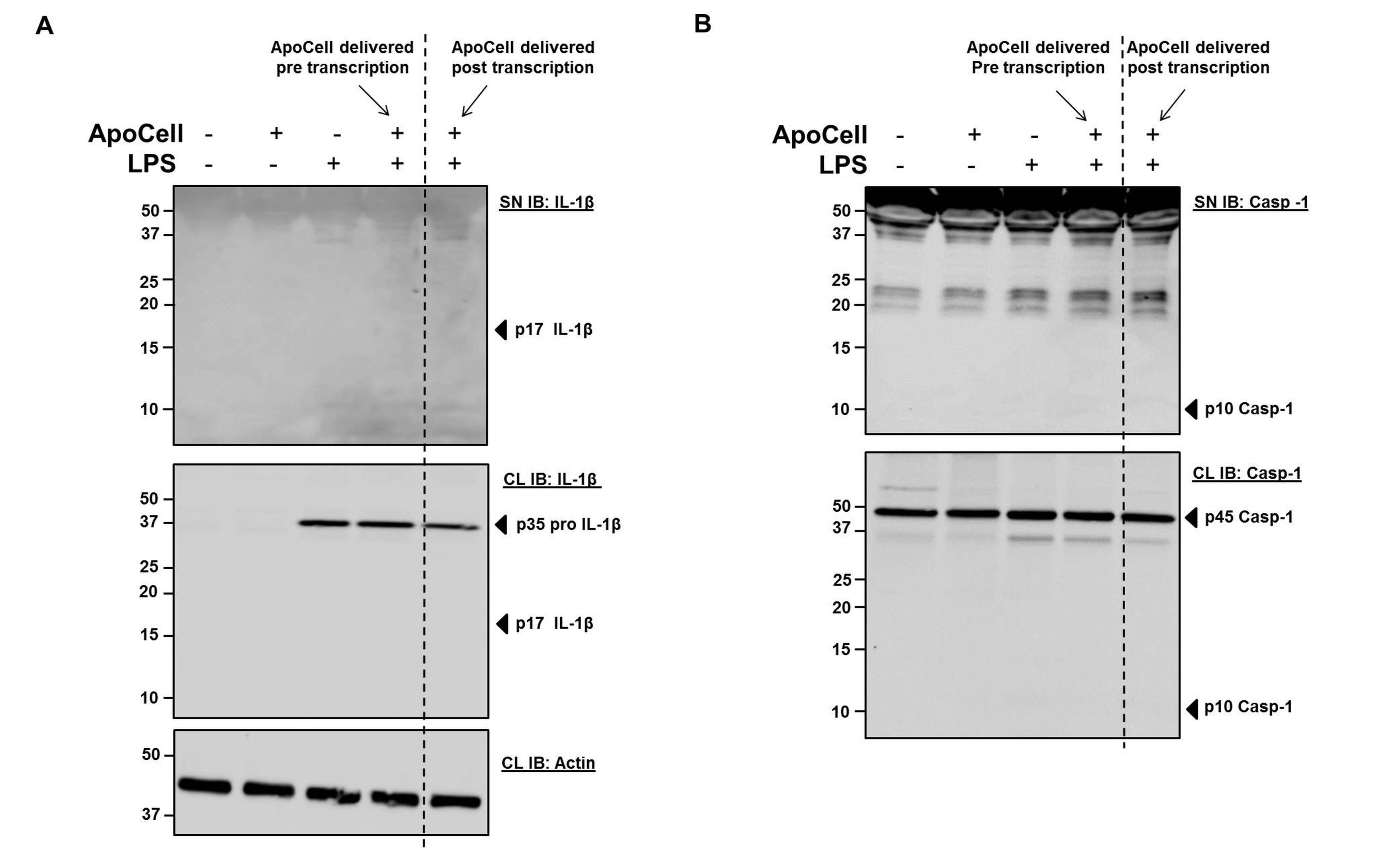
**

**Figure D. Influence of apoptotic cells and LPS on IL-1β and caspase-1 activation.** western blot analysis of IL-1β **(A)** and caspase-1 **(B)** in supernatant (SN) and cell lysate (CL) of pMΦ treated with apoptotic cells prior to LPS (prior to NF-κB signaling – pre-transcription, forth lane) or treated with LPS prior to apoptotic cell treatment (to promote NF-κB signaling – post-transcription, fifth lane, separated by dashed line). An anti-mouse actin served as a loading control. Shown are representative data of two experiments.

**
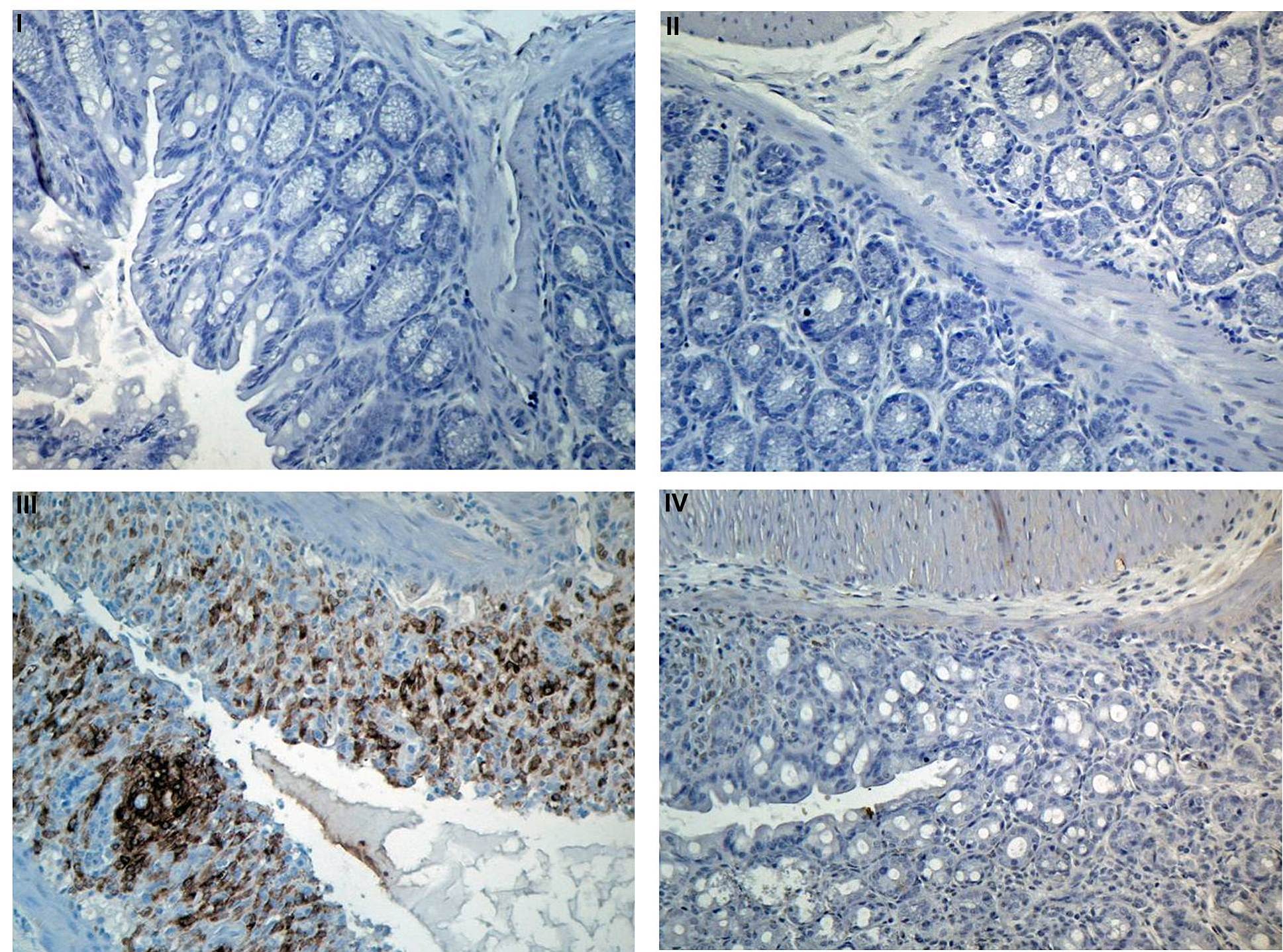
**

**Figure E.** Apoptotic cell treatment inhibits cyclooxygenase-2 (COX-2) in DSS-induced colitis. Mouse colon tissue sections were stained by immunohistochemistry assay using an antibody against mouse COX-2. After immunostaining, slides were counterstained by hematoxylin. All images are x200. **(I)** Staining control. Untreated colon stained with HRP-anti rabbit secondary antibody only without anti COX-2. **(II)** COX-2 expression in untreated colon (0% DSS+PBS). **(III)** COX-2 expression in 3% DSS treated colon (3% DSS+PBS). **(IV)** COX-2 expression in 3% DSS-treated colon with apoptotic cell infusion (3% DSS+ApoCell).

**
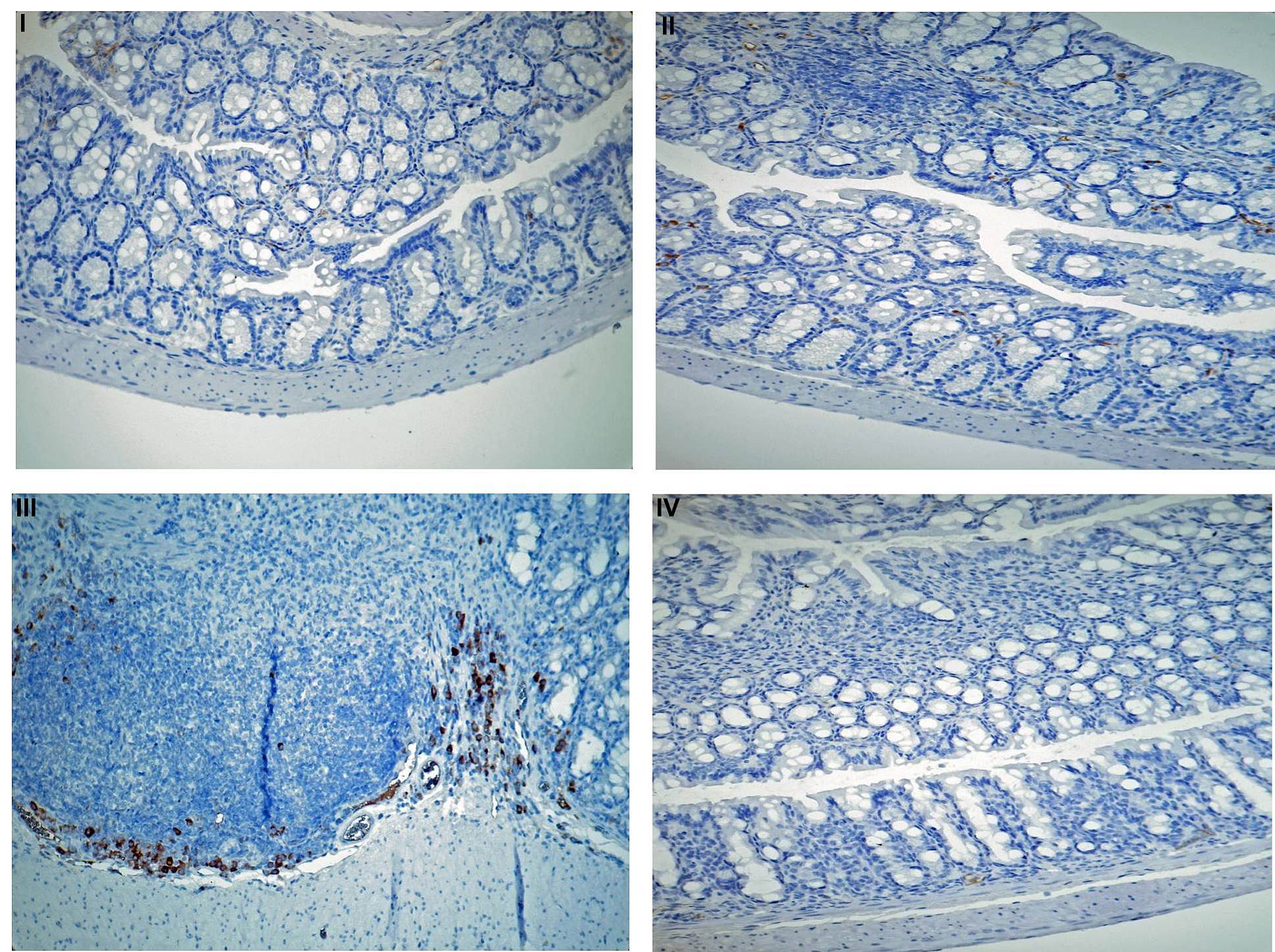
**

**Figure F.** **Apoptotic cell treatment inhibits Iκ-Bα phosphorylation in DSS-induced colitis.** Mouse colon tissue sections were stained by immunohistochemistry assay using an antibody against mouse phospho-Iκ-Bα (pIκ-Bα). After immunostaining, the slides were counterstained by hematoxylin. Images show pIκ-Bα. All images are x200. **(I)** Untreated colon stained with HRP anti-mouse secondary antibody only, without anti-pIκ-Bα. **(II)** pIκ-Bα staining in untreated colon (0% DSS+PBS). **(III)** pIκ-Bα expression in 3% DSS-treated colon (3% DSS+PBS). **(IV)** pIκ-Bα expression in 3% DSS treated colon with apoptotic cell infusion (3% DSS+ApoCell).
